# The effect of the whole-process care model of the medical union on the improvement of kinesiophobia and bone mineral density in patients with osteoporosis

**DOI:** 10.1186/s13018-024-04616-z

**Published:** 2024-02-27

**Authors:** Xiaoli Qian, Xiaodong Cao, Liyan Zhu, Xiaojuan Yao, Lina Gu, Xin Yu

**Affiliations:** 1 Department of Surgery, Wuxi People’s Hospital Affiliated to Nanjing Medical University, Wuxi, 214023 China; 2 Department of Nursing, Wuxi People’s Hospital Affiliated to Nanjing Medical University, Wuxi, 214023 China

**Keywords:** Bone density, Full care, Medical association, Osteoporosis, Phobia

## Abstract

**Objective:**

To observe the effect of the whole-process care model of the medical union on the improvement of kinesiophobia and bone mineral density in patients with osteoporosis.

**Methods:**

In this descriptive study, a convenient sampling method was used to select 148 patients with osteoporosis who visited the hospital from January 2020 to December 2021. Patients aged ≥ 18 years and diagnosed with osteoporosis through quantitative computed tomography (QCT) were included in the study. They were able to cooperate during follow-up and had normal cognitive function. Patients with combined spinal curvature, thoracic deformity, and pulmonary dysfunction, accompanied by severe cardiovascular or limb dysfunction, and those who withdrew midway or participated in other clinical studies were excluded. According to whether to use the whole-process care model of the medical union, they were divided into intervention group and control group, with 74 cases each. The control group used conventional care, and the intervention group used the whole-process care model of the medical association. The occurrence of kinesiophobia between the two groups were compared. The dual-energy X-ray absorption detector is used to measure differences in bone density changes.

**Results:**

There was no significant difference between the two groups in the TSK scale score and the incidence of kinesiophobia before intervention (*P* > 0.05). The TSK scale scores of patients in the intervention group were higher than those in the control group at 3 months and 6 months after operation (*P* < 0.05). The incidence of kinesiophobia in the intervention group for 3 months and 6 months was significantly lower than that in the control group (*P* < 0.05). There was no significant difference in bone mineral density between the two groups before and 3 months after intervention (*P* > 0.05). The bone mineral density of lumbar spine, femoral neck and total hip in the intervention group was significantly higher than that in the control group after 6 months of intervention (*P* < 0.05).

**Conclusion:**

The whole-process care model of the medical association is used for osteoporosis patients, which might reduce the risk of kinesiophobia and improve the bone density of the lumbar spine and total hip in patients. But further promotion and improvement of relevant support systems are needed to achieve comprehensive promotion and maximize clinical benefits in this field.

## Introduction

Osteoporosis is a systemic bone disease, which leads to bone thinning, thus increasing the risk of fracture. A prospective cohort study in Australia [1] found that early treatment of osteoporosis can reduce the risk of fracture in patients. Therefore, there is a need to improve nursing. Osteoporosis mainly affects middle-aged and elderly people. Osteoporosis induced fractures have become an important disease for the adult population living in developed economies in the Asia–Pacific region [2]. Kinesiophobia is an irrational fear of exercise, which is caused by fear of injury during exercise. Kinesiophobia is related to the decline of physical activity level. Patients who lack the knowledge related to osteoporosis are prone to kinesiophobia. It is even believed that physical activity also leads to the risk of falling or fracture [3]. A survey in China shows that the incidence of postoperative kinesiophobia in patients with osteoporotic vertebral compression fractures is 29.19% [4]. The medical association can combine the basic medical data of the hospital and the community. Because the medical resources of the hospital and the community are not effectively integrated in China, the medical consortium joint care mode is only applied to the chronic wound specialty in some large cities. There is no standardized or implementable medical association joint care scheme for the prevention, early identification and timely treatment of osteoporosis. It has not been effectively managed in primary medical associations. The whole-process care mode of the medical union is a medical treatment mode for osteoporosis patients designed on the basis of the medical association, which has the popularization and feasibility. The purpose of this study is to further determine the impact of the whole-process care model of the medical association on enhancing community residents' attention to osteoporosis, reducing the risk of kinesiophobia, and improving the bone mineral density (BMD) of patients. The care model and related results are as follows.

## Methods

### Research object

This study is a longitudinal study. A total of 148 patients with osteoporosis who visited the hospital from January 2020 to December 2021 were selected by convenient sampling method. They were divided into intervention group (*I*) and control group (*C*) according to whether to adopt the whole-process care mode of medical association, with 74 patients in each group. The calculation method for the required sample size is shown in formula (1).

1 $$n = \frac{{\sigma^{2} }}{{\Delta^{2} }}(Z_{\alpha /2} + Z_{\beta } )^{2}$$

In formula (1), $\alpha$ and $\beta$ represent the error probabilities in the two classification groups, with general values of 0.05 and 0.2. $\alpha$ represents the quantile function of a normal distribution. $\Delta$ represents the difference between two sets of numerical values. $\sigma$ represents the standard deviation, which is used to measure the volatility of numerical values. A large $\sigma$ indicates significant numerical fluctuations. Inclusion criteria are as follows: (1) Age ≥ 18, (2) osteoporosis was confirmed by quantitative computed tomography (QCT), and the diagnostic criteria refer to the Chinese QCT Guidelines for the Diagnosis of Osteoporosis [5], (3) 12-month follow-up, and (4) normal cognitive function. Exclusion criteria are as follows: (1) patients with kyphosis, thoracic deformity and pulmonary dysfunction, (2) the researcher withdrew from the group or participated in other clinical activities, and (3) sufferers with severe abnormalities of cardio-cerebrovascular or limb functions. The recruitment channel is to recruit osteoporosis patients database who visited the orthopedic department of a hospital from January 2020 to December 2021. Patients who meet the inclusion criteria (age, diagnostic criteria, follow-up period, etc.) are included in the study sample library for sampling. This study was reviewed by the Medical Ethics Committee of the hospital. The research content complies with the relevant research ethics standards of the Helsinki Declaration. The sex, age, education level, past medical history, frequency of physical exercise, type of chronic disease and severity of osteoporosis of patients are not statistically significant, with strong comparability (*P* > 0.05). This study is reviewed and approved by the Medical Ethics Committee.

### Nursing methods

#### Intervention group

On the basis of routine nursing, the whole-process care mode of medical association is adopted. (1) The research team is formed, consisting of 1 general practitioner, 1 head nurse and 5 responsible nurses from the community hospital of the medical association. General practitioners are responsible for setting the grading diagnosis and treatment plan of patients with osteoporosis. The head nurse is responsible for formulating a questionnaire on the factors related to kinesiophobia in terms of diagnosis, treatment, diet, exercise, medication and family support, and evaluating the characteristics of patients. The responsible nurse undertakes the specific nursing content.

(2) Patient characteristics are evaluated. According to the baseline survey of three medical communities in the early stage, the six dimensions of diagnosis, treatment, diet, exercise, medication and family support are analyzed. The possible influencing factors and prevention measures of each dimension are structured. ① In the “diagnosis and treatment”, the unknown disease, no medical treatment or no medical treatment are investigated. ② In the “medication”, it mainly includes the survey for awareness of calcium, vitamin D and commonly used anti-osteoporosis drugs. ③ In the “diet and exercise”, the knowledge of diet, scientific exercise, specific dietary requirements and exercise methods are collected. ④ The “family support” mainly focuses on the composition of family members, economic situation, whether there is social security, and the education level of individuals and family members.

(3) The improvement strategy is formulated. Brainstorming is used to formulate improvement strategies. ① In the “diagnosis and treatment”, general practitioners carry out graded diagnosis and treatment for patients in the community, and further implement the triage mechanism of medical association. ② In the “medication”, it is mainly improved through face-to-face communication, special lectures on osteoporosis and nurse supervision. It should also be explained in combination with the patient's complications. ③ In the “diet and exercise”, the home diet of patients is no longer a major problem through the formulation of osteoporosis diet manual. Most patients have insufficient knowledge of scientific exercise. Nurses supervise the patient's dietary structure referring to the Consensus of Chinese Experts on Rehabilitation Intervention for Primary Osteoporosis [6]. ④ In the “family support” dimension, the family support level of patients is improved by the synchronous management of patients and their families.

(4) Medical Association is mainly used for patients with mild osteoporosis (− 1.0 < *T* value < 1.0). Intervention is carried out through the WeChat groups and patient associations with the theme of osteoporosis treatment and care. Intervention types include functional exercise guidance and related knowledge lectures. Face-to-face communication, special lectures on osteoporosis, and special exhibition boards on osteoporosis explain or show the importance of proper exercise, methods and precautions of functional exercise, causes and hazards of kinesiophobia to patients and their families. The functional exercise can be performed by walking, fast walking or pedal exercise. It can be performed for 3 days a week and 20–30 min a day. The exercise time of 10–15 min is increased every 3 months, and the maximum continuous exercise time is 45 min. The responsible nurse is responsible for supervising the patient to complete the functional exercise, and the supervision time is 1 time/week. Lectures on diabetes, hypertension, hyperlipidemia, insulin, and osteoporosis drugs are held regularly to assess the medication habits of patients. Possible adverse reactions during medication need to be explained to reduce patients' concerns about medication.

(5) The linkage mode of medical association is mainly used for patients with moderate osteoporosis (− 2.5 < *T* value <− 1.0). The patients with moderate osteoporosis are mostly treated with drugs. The risk factors of osteoporosis, medication precautions, and prevention measures related to fracture are publicized and taught through expert sitting and professional interpreter explanation mode. The patient’s mobile phone sets the form of medication alarm clock to strengthen medication compliance. At the same time, those who have negative emotions and emotional experience should be timely dredged. After 3 months of medication, the osteoporosis of the patient is reviewed. If the patient becomes a mild patient, the patient is promptly adjusted to be a member of the medical association.

(6) The whole-hospital care is mainly aimed at patients with severe osteoporosis (*T* value ≤− 2.5 combined with fragile fracture). The diet, medication and functional exercise of patients are monitored daily during the hospital. An interactive meeting is held every 48 h, lasting for 60 min each time. The completion of the patient’s phased plan is evaluated. Those who complete the exercise plan should be encouraged. The uncompleted further analyzes the possible obstacles. The open questioning method is used to determine the patients’ own feelings about exercise and rehabilitation exercise, clarifying the risk of patients’ kinesiophobia. The severity of the disease is evaluated again after 3 months. The intervention strategy is further improved.

#### Control group

Routine nursing is implemented. The community health center sets up a special outpatient service for osteoporosis under the guidance of the superior hospital. Osteoporosis patients transferred from superior hospitals are accepted and health records were established. Under the guidance of general practitioners, patients are given routine medication, diet and life guidance. The medication guidance includes the medication mode, dosage and precautions during the medication of alendronate sodium tablets, alfacalcidol tablets and calcidol D. The diet guidance includes the distribution of osteoporosis diet manual, appropriate calcium and phosphorus ratio, sufficient calcium, vitamin AD, and various trace elements. Life guidance includes prevention of falls and proper waist and back muscle training. If the pain is severe, the community physician can give pain medication. Patients were told to avoid smoking, drinking, staying up late and other bad habits.

### Observation indicators

#### General data difference

The general data of patients are collected after enrollment, mainly including gender, age, education level, past medical history, frequency of physical exercise, type of chronic disease, and severity of osteoporosis.

#### Incidence of kinesiophobia

The patients are evaluated with the Tampa Scale of Kinesiophobia (TSK) before, 3 months after and 6 months after the intervention. The scale is prepared by Woby et al. [7]. There are 17 items in the scale, with 1–4 points for each item, representing strong disagreement to strong agreement (items 4, 8, 12 and 16 are scored reversely). The total score of the scale is 17–68. When the score is higher than 37 points, it is considered as having kinesiophobia. The value of Cronbach’s *α* is 0.778. The scale evaluators have all undergone TSK professional scale testing training. After passing the assessment, they will be on duty to participate in this study. Two scale evaluators jointly complete the scale evaluation work.

#### Bone mineral density

The BMD of lumbar spine, femoral neck and total hip of patients in the two groups are measured by dual-energy X-ray absorptiometry before and 3 and 6 months after the intervention. It is measured by a professional technician who measured BMD in the hospital. The phantom test is carried out before the measurement. The CV value of the three measuring positions is 1%. During the measurement, the patient's feet were fixed and the patient's femur is required to rotate inward. The average BMD value of each part is taken for comparison after three measurements. When T value ≤− 2.5, it is considered as osteoporosis patient [8].

#### Self-perceived burden scale

They are assessed with Self-Perceived Burden Scale (SPB) before and 3 and 6 months after the intervention. The scale adopts 1–5 grade scoring method. The score includes 1–4 points. The total score is less than 20, indicating that the patient has no burden. A score of 20–29 indicates that the patient has a slight feeling burden. A total of 30–39 points indicate that the patient has moderate feeling burden. A score of ≥ 40 indicates that the patient has a heavy feeling burden. The Cronbach’s *α* coefficient of the scale is 0.890. The validity value KMO is 0.950.

#### Quality of life scale

Social role, physiological role, emotional role, physical state as well as mental state in the SF-36 questionnaire are used for statistics. Each dimension contains 2–10 items, and the items are A–J. The possible scoring results of each dimension are shown in Table 1.

**Table 1:** Possible Scores of Each Dimension

Dimension	Accumulation calculation method	Possible score		
		Range	Highest	Minimum
Physiological function	4(A + B + C + D)	4	8	4
Physical condition	7 + 8	9	11	2
Social function	6 + 10	8	10	2
Psychological status	9(B + C + D + F + H)	25	30	5
Emotional function	5(A + B + C)	3	6	3

### Statistical analysis of experimental data

SPSS 24.0 software is used for data processing. The normal measurement data and counting data are expressed by *x* ± *s* and frequency, respectively. The independent sample *t* test and *χ*^2^ test are used for inter-group comparison. The measurement data at different time points within the group are analyzed by repeated measurement variance. When *α* = 0.05 and *P* < 0.05, the difference is significant.

## Results

### Comparison of general data

There was no statistically significant difference between the two groups in gender, age, education level, past medical history, physical exercise frequency, chronic disease type, and osteoporosis severity (*P* > 0.05). Table 2 describes the specific information of patients.

**Table 2:** Comparison of general data

Group (*n*)	Gender		Age (years)	Education level (cases)		Physical exercise frequency (times/week)	
	Man	Female		Junior high school and below	High school and above	< 3	≥ 3
*I* (74)	12	63	58.28 ± 4.33	39	35	40	34
*C* (74)	13	61	59.35 ± 4.04	36	38	39	35
*χ*^2^/*t*	0.199		− 1.551	0.242		0.027	
*P*	0.656		0.123	0.623		0.870	

Group	Severity of osteoporosis (cases)			Types of chronic diseases (cases)			
	Mild	Moderate	Severe	Diabetes mellitus	Hypertension	Hyperlipidemia	Without
*I* (74)	25	17	32	21	14	19	20
*C* (74)	21	21	32	22	13	15	24
*χ*^2^/*t*	0.769			0.895			
*P*	0.681			0.827			

### Comparison of kinesiophobia scores

Before intervention, there was no statistically significant difference in the TSK scale scores between the two groups (*P* > 0.05). The TSK scale scores of patients in the group I were higher than those in the C at 3 months and 6 months after operation, with statistical significance (*P* < 0.05). Overall, the interaction between groups, time and time was statistically significant (*P* < 0.05). Table 3 describes patient specific information.

**Table 3:** Comparison of TSK scores

Group	Before intervention	3 Months after intervention	6 Months after intervention	*F*	*P*
*I* (74)	48.42 ± 5.02	41.01 ± 3.75	35.20 ± 3.01	*F*inter-group = 32.52	*P*inter-group < 0.001
*C* (74)	48.85 ± 4.23	45.62 ± 4.85	38.88 ± 5.11	*F*time = 341.08	*P*time < 0.001
*t*	− 0.599	− 6.470	− 5.331	*F*between = 12.02	*P*between < 0.001
*P*	0.550	< 0.001	< 0.001		

### Comparison of changes in the incidence of kinesiophobia

**Fig. 1:**
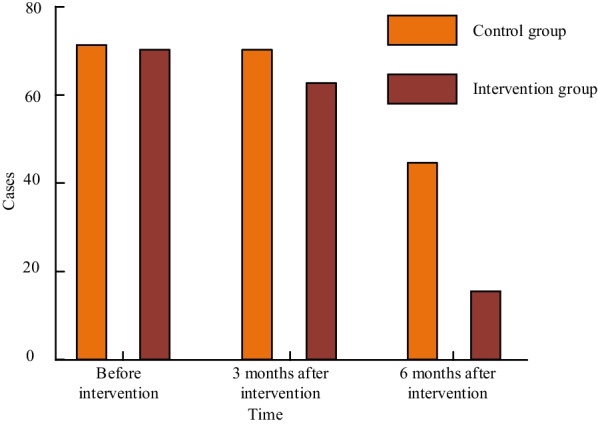
Comparison of Changes in the Number of cases of Kinesiophobia

Figure 1 shows the number of patients with phobia in the two groups. From Fig. 1, the incidence of kinesiophobia in both groups showed a gradual downward trend. The main difference was more obvious at 6 months after intervention.

The incidence of kinesiophobia before intervention was 100.00% and 97.30%, respectively, and they were not significant (*P* > 0.05). After 3 and 6 months of intervention, the incidence of kinesiophobia in the group I was lower than that in the C, and the difference was statistically significant (*P* < 0.05). Table 4 describes patient specific information.

**Table 4:** Comparison of changes in the incidence of kinesiophobia [cases (%)]

Group	Before intervention	3 Months after intervention	6 Months after intervention
*I* (74)	72 (97.30)	64 (86.49)	16 (21.62)
*C* (74)	74 (100.00)	72 (97.30)	46 (62.16)
*χ*^2^	2.014	5.765	24.812
*P*	0.156	0.016	< 0.001

### Comparison of bone mineral density changes

The results of BMD at lumbar spine before and after intervention in two groups are shown in Table 5. They were no significant difference in BMD of lumbar spine between the two groups before and 3 months after intervention (*P* > 0.05). After 6 months of intervention, the bone density of the lumbar spine in the group I was higher than that in the *C*, and the difference was statistically significant (*P* < 0.05). Inter-group and time effects were significant in lumbar BMD (*P* < 0.05).

**Table 5:** Comparison of bone mineral density at lumbar spine ($\overline{x} \pm s$, g cm^−2^)

Group (*n*)	Before intervention	3 Months after intervention	6 Months after intervention	*F*	*P*
*I* (74)	0.74 ± 0.12	0.75 ± 0.12	0.80 ± 0.10	*F*inter-group = 4.183	*P*inter-group = 0.043
*C* (74)	0.73 ± 0.10	0.74 ± 0.11	0.76 ± 0.10	*F*time = 6.911	*P*time = 0.001
*t*	0.872	0.657	2.166	*F*between = 0.492	*P*between = 0.612
*P*	0.385	0.512	0.032		

The BMD results at the femoral neck before and after the intervention in the two groups are shown in Table 6. There was no significant difference in BMD at the femoral neck between the two groups before and 3 months after the intervention (*P* > 0.05). After 6 months of intervention, the bone density of femoral neck in the study group was higher than that in the *C* (*P* < 0.05). Time effect was significant in BMD of femoral neck (*P* < 0.05).

**Table 6:** Comparison of bone mineral density at femoral neck (*x* ± *s*, g cm^−2^)

Group (n)	Before intervention	3 months after intervention	6 months after intervention	*F*	*P*
*I* (74)	0.60 ± 0.09	0.60 ± 0.08	0.57 ± 0.06	*F*inter-group = 0.030	*P*inter-group = 0.863
*C* (74)	0.58 ± 0.09	0.59 ± 0.07	0.56 ± 0.07	*F*time = 3.990	*P*time = 0.022
*t*	0.843	− 1.047	0.428	*F*between = 1.062	*P*between = 0.349
*P*	0.400	0.297	0.669		

The BMD results at the total hip of the two groups before and after intervention are shown in Table 7. There was no statistically significant difference in total hip BMD between the two groups before and 3 months after intervention (*P* > 0.05). After 6 months of intervention, the bone density of the total hip in the study group was higher than that in the *C*, with a statistically significant difference (*P* < 0.05). Time effect, inter-group and time interaction had statistical significance in total hip BMD (*P* < 0.05).

**Table 7:** Comparison of Total Hip Bone Mineral Density ($\overline{x} \pm s$, g cm^−2^)

Group (*n*)	Before intervention	3 Months after intervention	6 Months after intervention	*F*	*P*
I (74)	0.72 ± 0.08	0.68 ± 0.23	0.80 ± 0.16	*F*inter-group = 0.921	*P*inter-group = 0.340
C (74)	0.69 ± 0.08	0.71 ± 0.09	0.74 ± 0.08	*F*time = 16.369	*P*time < 0.001
*t*	1.266	− 1.250	2.732	*F*between = 4.732	*P*between = 0.009
*P*	0.207	0.214	0.007		

### Quality of life assessment

The quality of life evaluation before and after treatment is shown in Table 8. There was no significant difference in the scores of the two groups in each dimension before treatment (*P* > 0.05). After 3 months of treatment, the scores of the two groups in all aspects were different (*P* < 0.05). After 6 months of treatment, the difference between each dimension was more significant (*P* < 0.05).

**Table 8:** Evaluation of quality of life

Dimension	Treatment time	*I*	*C*	*t*	*P*
Social function	Before treatment	46.22 ± 5.03	47.23 ± 5.07	0.258	> 0.05
	3 Months after treatment	75.03 ± 4.78	69.34 ± 5.32	4.035	< 0.05
	6 Months after treatment	78.06 ± 4.65	70.13 ± 4.27	4.301	< 0.05
	*t*	9.405	6.875	–	–
	*P*	< 0.05	< 0.05	–	–
Physiological function	Before treatment	49.21 ± 5.33	49.04 ± 4.82	0.140	> 0.05
	3 Months after treatment	76.92 ± 5.43	63.74 ± 4.17	4.752	< 0.05
	6 Months after treatment	79.28 ± 5.91	68.34 ± 4.76	4.094	< 0.05
	*t*	8.874	2.351	–	–
	*P*	< 0.05	< 0.05	–	–
Emotional function	Before treatment	49.08 ± 5.88	50.17 ± 4.96	0.453	> 0.05
	3 Months after treatment	80.86 ± 6.02	73.44 ± 5.89	4.854	< 0.05
	6 Months after treatment	84.23 ± 5.73	78.46 ± 4.73	4.316	< 0.05
	*t*	9.102	6.840	–	–
	*P*	< 0.05	< 0.05	–	–
Physical condition	Before treatment	50.14 ± 5.52	50.17 ± 5.61	0.072	> 0.05
	3 Months after treatment	81.33 ± 5.51	71.43 ± 5.57	4.106	< 0.05
	6 Months after treatment	84.31 ± 42	75.89 ± 5.06	4.612	< 0.05
	*t*	9.532	6.779	–	–
	*P*	< 0.05	< 0.05	–	–
Psychological status	Before treatment	55.22 ± 5.43	55.19 ± 5.50	0.026	> 0.05
	3 Months after treatment	83.96 ± 5.45	76.22 ± 5.11	6.304	< 0.05
	6 Months after treatment	85.13 ± 4.76	80.48 ± 5.72	5.46	< 0.05
	*t*	8.963	5.650	–	–
	*P*	< 0.05	< 0.05	–	–

### Comparison of self-perceived burden

Table 9 shows the patients’ SPB before and after treatment. Before treatment, there was no significant difference between the two groups in terms of SPB. After 3 months of treatment, the difference between the two groups was small. After 6 months of treatment, there was a significant difference between the two groups in the number of cases with no significant pressure.

**Table 9:** Comparison of Self-perceived burden (*n* = 148)

Project	Treatment time	*I*	Proportio*n* (%)	*C*	Proportio*n* (%)
No obvious burden	Before treatment	7	4.7	12	0.08
	3 Months after treatment	19	12.8	19	12.1
	6 Months after treatment	54	36.5	32	21.7
Light burden	Before treatment	20	13.5	20	13.5
	3 Months after treatment	30	20.2	28	18.9
	6 Months after treatment	46	31.1	30	20.3
Moderate burden	Before treatment	36	24.3	32	21.6
	3 Months after treatment	42	28.3	31	20.9
	6 Months after treatment	31	20.9	44	29.7
Heavy burden	Before treatment	85	57.4	84	56.8
	3 Months after treatment	55	37.1	70	47.3
	6 Months after treatment	17	11.5	42	28.4

## Discussion

Osteoporosis is a chronic degenerative disease that requires continuous disease management [9]. The community health service unit and the general hospital carry out a three-level prevention project centered on the medical association. A two-way referral is established to lay the foundation for the continuous disease management of osteoporosis patients. WHO has developed a comprehensive treatment framework for osteoporosis and improved secondary fracture prevention through standardized nursing and health consultation. However, this plan has not been fully implemented, especially for the prevention and control effect of bone loss [10]. Oksuz et al. [11] believe that reasonable exercise can reduce the kinesiophopia and the pain in patients with osteoporosis, and improve the quality of life. Kinesiophopia is a clinical manifestation of fear in patients toward movement or daily activities due to their own chronic pain. Kinesiophopia is prone to have adverse effects on the daily life, and even cause disability [12]. However, no effective intervention plan has been formed for kinesiophopia in clinic.

The whole-process care mode of the medical association is a new mode of graded diagnosis and treatment and continuous care for osteoporosis patients, which is built based on the graded diagnosis and treatment management method of the medical association. Unlike the operation mechanism of medical services in the USA, China’s medical consortia is not dominated by market-oriented regulation, but a referral model dominated by disease diagnosis and treatment [13]. At the same time, the whole-process care mode is combined with the hierarchical diagnosis and treatment management of the medical association to effectively meet the continuous disease management needs of osteoporosis patients. Yang et al. [14] have established a hierarchical diagnosis and treatment management model with osteoporosis as the main body, which is completed in Ruijin-Luwan Medical Union. It provides a new idea for improving the quality of life of osteoporosis patients. However, it is not known whether the whole-process care mode of the medical association can provide support for BMD improvement of osteoporosis patients.

The results of this study confirmed that the whole-process care model of the medical association can effectively reduce the kinesiophopia in patients with osteoporosis and the risk of occurrence. According to the data, the intervention begins to take effect three months after the intervention. Routine nursing pays attention to medication guidance and diet guidance. Analgesics are used to control pain. From the actual effect, the patients with osteoporosis still have kinesiophopia. The patients in the group I were first divided into three grades: mild, moderate and severe. Different intervention routes were selected for different grades of patients to reduce unnecessary operations for some patients. At the same time, the group I applied the patient association of medical association, the medical association linkage mode, and the whole-process care. The patient association of the medical association is designed and developed with reference to peer support technology, which can strengthen the benefits of functional exercise for patients with mild osteoporosis, extend the functional exercise time, and improve the awareness for kinesiophopia. The medication knowledge of diabetes, hypertension, hyperlipidemia and other chronic diseases closely related to osteoporosis is included in the management category, so as to reduce the medication errors of patients and promote the medication compliance of anti-osteoporosis drugs [15]. The medical association linkage model aims to improve the kinesiophopia behavior of moderate osteoporosis. Most patients with moderate osteoporosis have serious drug compliance problems. Affected by their own pain symptoms, there are a variety of adverse emotions [16]. Therefore, expert consultation, professional interpreter explanation, setting medication alarm clock, relieving adverse emotions and other measures are taken as intervention programs to improve medication compliance and adjust adverse emotions. The purpose of whole-hospital care is to improve the kinesiophopia behavior of patients with severe osteoporosis treated in the hospital. As the supervisor of medication, diet and functional exercise, the nursing staff formulates individualized and continuous exercise plans, explores the root causes of patients' kinesiophobia, and establishes improvement strategies. This research idea is based on the disease management scheme advocated by the Expert Consensus on the Interpretation of the Grading Diagnosis and Treatment Policy of Osteoporosis and the Scheme [17]. Thus, the whole-process care model of the medical association can effectively improve the kinesiophobia and the osteoporosis risk.

After further comparison of BMD, the whole-process care mode of the medical association can effectively improve the BMD level of the lumbar spine, which may be related to the reduction of the patient's kinesiophobia. Lack of exercise is one of the main reasons for bone loss [18]. Resistance exercise and aerobic exercise can avoid bone loss [19]. In this study, the patients with osteoporosis receive different diagnosis and treatment, as well as interventions in their diet, medication, exercise, and family support. The coordination function of the medical association to the community health resources and the hospital helps patients to reasonably choose the medical channels. At the same time, patients can get more adequate drug intervention and functional exercise supervision during their stay at home. In the functional exercise, it is simple and convenient to walk or pedal. The Expert Consensus on Nutrition and Exercise Management of Patients with Primary Osteoporosis [20] emphasizes that patients with osteoporosis should have at least 150–300 min of moderate intensity training every week, which needs to be continued. Walking is more acceptable for middle-aged and elderly people, and the injury risk is lower. However, short-term walking does not increase the bone density of lumbar spine and femoral neck. Exercise lasting for 6 months can increase the bone density of female femoral neck. When walking and jogging reach enough high mechanical stress, they will produce reaction force on the bone mass ground to stimulate the increase of bone mass and solve the poor bone density [21]. From the results, it is consistent with the trend of BMD change in 6 months. This result is basically consistent with the results of Ma et al. [22]. This may be because for BMD of the femoral neck, various exercise interventions significantly increase bone density compared to insufficient exercise. The best type is physical and mental exercise [23]. The bone density of the body mainly affects the hip, while it has a slight impact on the knee joint [24]. The full care model of the medical consortium intervenes in the continuity of patient movement through multiple aspects such as family and society. It provides personalized care plans for patients with different degrees of osteoporosis, effectively improving the insufficient hip bone density in patients. However, there is no stratified analysis for age in this study. Therefore, it is not possible to determine whether the results of this study are applicable to all age groups of patients. The study will further integrate the graded drug use management and exercise management program of the medical association, and provide scientific basis for more age groups of patients with osteoporosis.

## Conclusion

To sum up, the whole-process care model of the medical association for osteoporosis patients can reduce the risk of kinesiophobia and improve the bone density of the lumbar spine, femoral neck and total hip of patients. It is worth promoting. In the future, the medical consortium model can be incorporated into the clinical pathway and standards for osteoporosis management, and applied to hospitals at all levels. A professional osteoporosis medical consortium team has been established, with clear roles and tasks for each member. The osteoporosis medical consortium management information system has been developed to achieve data sharing and process management. A complete patient tracking management standard procedure has been developed to ensure smooth handover at different stages.

## Data Availability

All data generated or analyzed during this study are included in this published article.
